# Nursing Homes and Our "Approved Homes"

**Published:** 1912-10-19

**Authors:** 


### The Editor's Letter-Box.

### Nursing Homes and Our "Approved Homes."

*To the Editor of* The Hospital.

Sir,—I am so glad to see you are in favour of mending, and not ending, nursing homes. I wish also to thank you for your kind remarks in August 17 issue, and to assure you I am a very practical and not at all an imaginative person, and that I wrote wholly from intimate knowledge of about half a dozen such actual nursing homes as described; that each of the managers I count amongst my friends; that we are, perhaps, a set of idealists trying to realise our ideals, but are also large-minded enough to think and hope we are not an exclusive set and the only one of its kind.

That there exist a number of so-called "nursing homes" that all of us high-souled women—and men—would like to see ended, that are in some cases a disgrace to the profession, and in others a scandalous filching of its name, that are used for commercial gain merely, and for worse, we know from first-hand report; but personal acquaintance with such is *nil.* To give you an instance: A trained nurse accepted a post in one by letter; she arrived, and was introduced to a number of men lounging in dressing-gowns in their bedrooms and out of them, none of whom appeared ill enough to need a nurse, and one of whom took the head of the table at dinner so attired, having been "at his office" all day. There were no other women besides the superintendent seen; there was an unsolved mystery of a skeleton key, and no lock on the nurse's bedroom door. She decided before night that this was no place for her, paid a month's salary in lieu of notice, and left. Her action was taken as coolly as if it had been an everyday occurrence. On consulting a doctor of the town on the subject, he remarked it was a very serious thing to condemn a nursing home. Now, taking the mildest view of appearances, they were those of a badly conducted house, to say the least of it, and unworthy of support without reformation.

Your system of "Approved Homes" and co-operation between homes themselves and homes and patients is excellent. It now only needs the hearty support of the medical profession, given unstintingly to the good ones and its utter ignoring, if not condemnation, of the bad ones, to prove what are worthy and that they are needed.

Meta.

